# Platelet Thromboembolism after Lung Transplantation

**DOI:** 10.1155/2009/650703

**Published:** 2009-05-25

**Authors:** Carles Bravo, Joaquim Majó, Fernando Ruiz, Laura Muntaner, Víctor Monforte, Joan Solé, José Maestre, Ferran Morell, Antonio Roman

**Affiliations:** ^1^Department of Pneumology, Hospital Universitari Vall d'Hebron, Universitat Autònoma de Barcelona, Passeig Vall d'Hebron 119-129, 08035 Barcelona, Spain; ^2^CIBER de Enfermedades Respiratorias (CIBERES), Instituto de Salud Carlos III, Madrid, Spain; ^3^Department of Pathology, Freeman Hospital, Newcastle upon Tyne, UK; ^4^Department of Internal Medicine, Hospital Universitari Vall d'Hebron, Barcelona, Spain; ^5^Department of Thoracic Surgery, Hospital Universitari Vall d'Hebron, Barcelona, Spain

## Abstract

Lung transplant patients have an increased risk of pulmonary embolism which is often associated with hypercoagulability disorders. We present a case of sudden death resulting from pulmonary intravascular platelet thromboembolism following a single-lung transplant.

## 1. Case Report

A 60-year-old woman was diagnosed with pulmonary fibrosis-like nonspecific interstitial pneumonia in 2004 by surgical lung biopsy which revealed advanced pulmonary fibrosis and morphological changes in pulmonary hypertension. Clinically, she presented functional class III dyspnea according to the NYHA classification. Respiratory function showed forced vital capacity (FVC) of 1.51liters (51% of predicted), forced expiratory volume in 1 second (FEV1) of 1.261 liters (58% of predicted), and FEV1/FVC of 84%. The echocardiographic study did not show any signs of pulmonary hypertension. During her progress she suffered two episodes of spontaneous pneumothorax, requiring drainage and pleural abrasion. She was diagnosed with Sjögren's syndrome (SS) by salivary gland biopsy owing to dry mouth and scapular belt pain. As part of the patient's routine rheumatological care, a full immunoglobulin antibody screen showed precipitating antibodies to the extractable nuclear antigens Ro/SS-A. Other immunological tests including cryoglobulins, lupus anticoagulant, antiphospholipid antibodies, and rheumatoid factor were repeatedly negative prior to transplantation. Her medical history included systemic arterial hypertension, hypertensive cardiomyopathy, and severe asymptomatic diastolic dysfunction with conserved left ventricle function.

The patient was evaluated for a lung transplant in January 2005 and, in March of the same year, was placed on the active waiting list for lung transplantation. A right single-lung transplant was performed in December 2005, with no intraoperative complications. Extracorporeal circulation was not required and evolution in the intensive care unit was satisfactory, with extubation at 24 hours. Bilateral pleural drainage was inserted with scant serohematic production. Subcutaneous low-molecular-weight heparin (LMWH) was started at that time at a dose of 60 mg every 24 hours. On the fourth day posttransplant, she was transferred to the pneumology ward where she remained afebrile and hemodynamically stable. Blood analysis showed hematocrit 29%, platelets 142000, prothrombin time 103%, normal liver and kidney function Na+: 141 mmol/L, K+: 4.7 mmol/L.

Immunosuppressant treatment with i.v. 6-methyl-prednisolone, cyclosporine A and azathioprine was started, together with prophylaxis against opportunistic infections with nebulized amphotericin B and i.v. ganciclovir. Posttransplant ventilation/perfusion scintigraphy showed good perfusion of the transplanted right lung (79.5%), with a minimal defect in the middle lobe and global hypoperfusion of the native left lung (20.5%). The patient's general status was good, with occasional dry cough attacks. Room air oxygen saturation was 94–96%. On the night of the 6th postoperative day she presented severe pain in the right hemithorax and sudden dyspnea, needing an increase in oxygen requirements. A chest X-ray performed yielded no significant pathologic findings. She was maintained with saturations of 92-93% with high oxygen flow until she presented cutaneous cyanosis, severe irreversible desaturation, and cardiorespiratory arrest.

Orotracheal intubation was started with assisted ventilation and cardiopulmonary resuscitation. The monitor tracing showed atrioventricular blockage; an external pacemaker was placed without success. Pericardiocentesis performed ruled out effusion or pericardiac tamponade. Ventricular fibrillation later appeared which was not reversed after defibrillation and adrenaline and atropine administration. The patient died after more than one hour of resuscitation measures.

**Figure 1:**
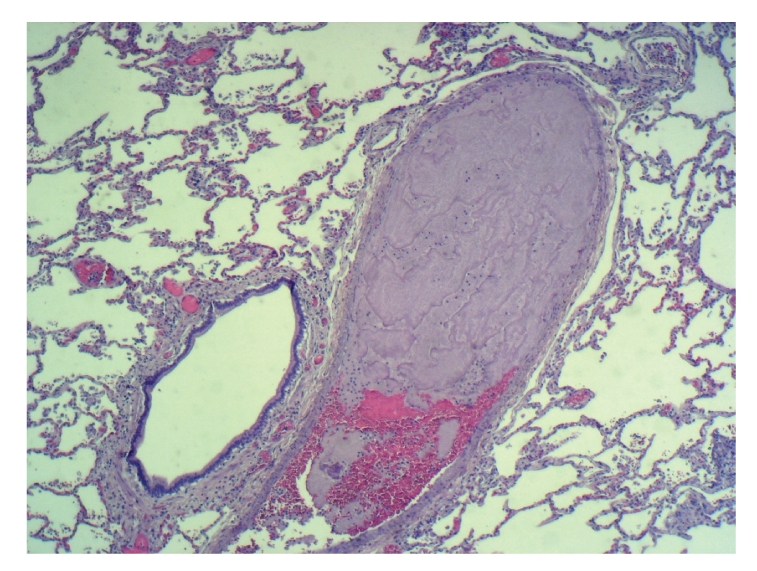
Moderate dilatation of the lumen of a preacinar artery (comparing its diameter with that of the adjacent bronchiole) that is occluded by clear eosinophilic material arranged in bands, and without red blood cells in its distal portion. Hematoxylin-eosin, x25.

**Figure 2:**
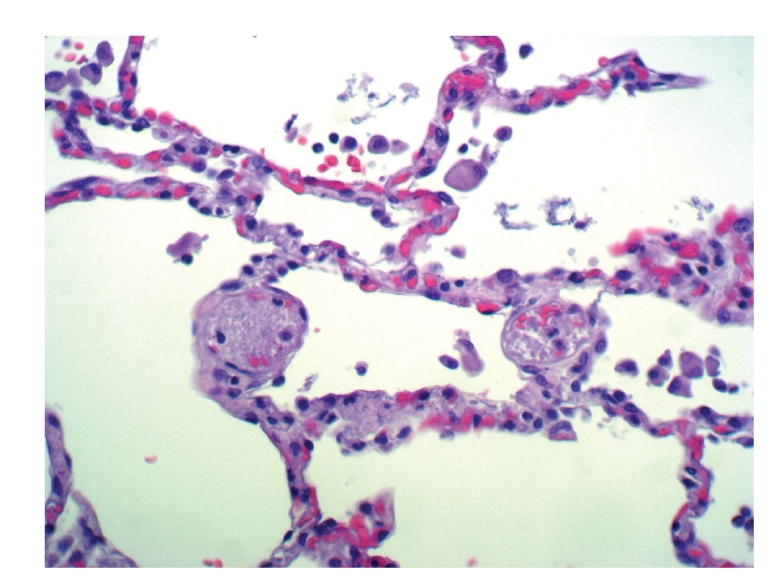
Pulmonary filed at medium magnification showing two intra-acinar vessels dilated by numerous puntiform, anucleated eosinophilic structures, much smaller in size than those of red blood cells. Hemotoxylin-eosin, x100.

**Figure 3:**
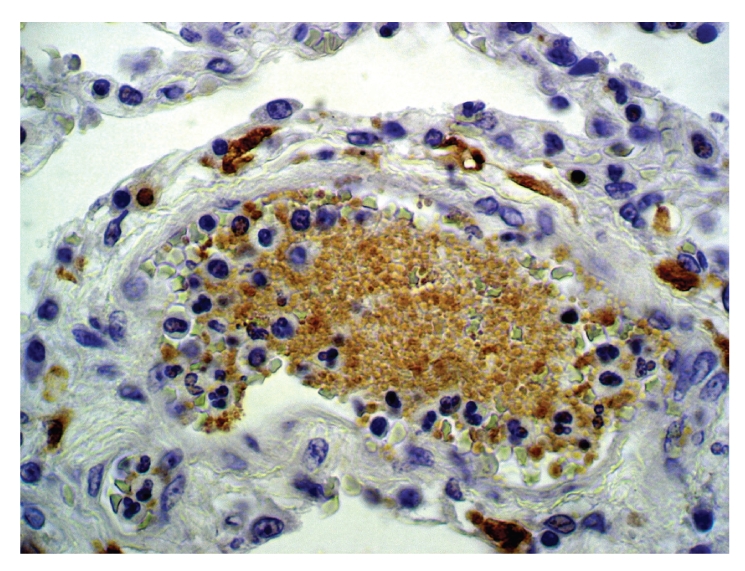
Immunohistochemical staining with Factor XIIIa staining the intravascular anucleated structures, showing the platelet origin of the thrombus. Immunohistochemistry, Factor XIIIa, x250.

A clinical autopsy requested showed multiple platelet thromboemboli (Figures 1, 2, and 3) dilating preacinar vessels, intraacinar vessels, and alveolar capillaries of both lungs, though primarily affecting the grafted right lung.

## 2. Discussion

Infections, acute and chronic rejection, and diffuse alveolar damage have been described as frequent causes of death in lung transplant recipients [1–3]. In the early posttransplant period, other leading factors limiting survival in these patients include primary graft failure, surgical complications, and pulmonary hemorrhage. The prevalence of venous thromboembolism (VTE) which includes deep vein thrombosis (DVT) and pulmonary embolism (PE) in lung transplant recipients has not been well established and few data are available on the contribution of PE to acute mortality and morbidity in these patients. Some authors have reported that these patients have an increased risk of VTE, which would vary between 8% and 22% according to the series. VTE is often associated with an increased amount of vascular trauma, higher levels of immunosuppression, use of cyclosporine, hypercoagulability disorders, need for cardiopulmonary bypass, longer ischemic time, older age, female sex, cytomegalovirus disease, diabetes mellitus, pneumonia, rejection, central venous catheters, and recipients with idiopathic pulmonary fibrosis. The majority of VTEs occur within the first month to 3 months postoperatively [4–8].

Most cases, like ours, are diagnosed at autopsy; thus, PE may be an underrecognized complication contributing to respiratory failure in the early post-lung transplant period. Burns and Iacono [6] studied 126 autopsies of lung and heart-lung transplant recipients and found a high prevalence of PE (27%), especially in the early postoperative period and in mechanically ventilated patients. Although PE was a frequent finding on autopsy examination, it was a rare primary cause of death (6.8%). PE more often affected single- and double-lung recipients compared with heart-lung recipients and preferentially affected the allograft.

This patient was diagnosed of SS, an autoimmune disease with symptoms and serological findings often overlapping with systemic lupus erythematosus (SLE). Thromboembolic events are common in SLE, but not in SS [9]. However, case reports have been described in SS patients who developed fulminant multiorgan disease due to thrombotic diathesis [10]. Associations between SS and the presence of anthiphospholipid antibodies have been reported. These antibodies play a role in thrombosis by an effect on platelet membranes, endothelial cells, and clotting protein such as prothombin, protein C, and protein S. Patients with antiphospolipid syndrome (APS) may develop a broad spectrum of pulmonary disease like PE, pulmonary hypertension, microvascular pulmonary thrombosis, pulmonary capillaritis, and alveolar haemorage [11]. This association might be a remote possibility in our case since, on the one hand, the patient had a prior negative full immunoglobulin antibody screen except for anti-Ro/SS-A and, on the other hand, the patient never had any thromboembolic complication before the lung transplant. Obviously, in these patients when considering the risk factors for thromboembolism, a full immunoglobulin antibody screen prior to transplantation must be done.

Pathologic findings in our patient, with the presence of multiple platelet thromboembolism in pulmonary vessels, differ clearly from those of typical PE. To our knowledge, this histologic finding has never been described previously in a lung transplant recipient.

Massive platelet thromboembolism is a possible cause of death in patients dying unexpectedly following recent liver transplantation [11, 12]. The cause of this extensive platelet activation in liver transplant recipients is uncertain and may be multifactorial. Nontransplant patients who showed these changes had a known coagulopathy or recognized risk of embolic disease. In any event, and unlike our case, those authors were able to show patent platelet consumption antemortem in some of these patients, probably due to factors such as hemorrhage or disseminated intravascular coagulation, requiring in some cases platelet concentrate transmission. 

One possibility is that release of tissue thromboplastin or cell debris from the ischemic donor liver may activate platelet aggregation. Similarly, cross-clamping of the aorta during emergency surgery has been shown to lead to a raised incidence of pulmonary microembolism. Experimental procedures in animals cause a significant fall in serum fibrinogen and result in some fibrin and numerous platelet thrombi and aggregates within the lungs as a result of trauma to the endothelium and stasis [13]. Probably during lung transplantation, hypoxia, metabolic acidosis, chronic lung disease, and several mechanisms of platelet aggregation may act synergistically and result in endothelial damage and local activation of platelet aggregation in the lungs despite prophylaxis with LMWH. Unfortunately, this phenomenon may only become clinically evident when it causes severe respiratory disease or death. 

Finally, it should be emphasized that the diagnosis in our case was made following examination of necropsic material. Histologically, the characteristic involvement of low-caliber pulmonary vessels suggests that the process could be diagnosed in some cases by detailed study of the transbronchial biopsy. Nevertheless, we believe that the rapid onset of the clinical picture, as occurred in our patient, hardly supports the usefulness of this invasive technique. 

We present a case of sudden death resulting from pulmonary intravascular platelet thromboemboli following a single-lung transplant in a patient with pulmonary fibrosis and SS who received adequate prophylaxis with LMWH. Therefore, we believe that platelet thromboembolims should be considered among the causes of sudden death early after lung transplantation.
